# Diabetic retinopathy, visual impairment and ocular status among patients with diabetes mellitus in Yemen: A hospital-based study

**DOI:** 10.4103/0301-4738.53055

**Published:** 2009

**Authors:** Mahfouth A Bamashmus, Abdallah A Gunaid, Rajiv B Khandekar

**Affiliations:** Department of Ophthalmology, Faculty of Medicine and Health Sciences, Sana'a University and Ibn Al-Haitham Eye Center, University of Science and Technology, Oman; 1 Department of Medicine, Faculty of Medicine and Health Sciences, Sana'a University and Ibn Al-Haitham Eye Center, University of Science and Technology, Oman; 2 British Columbia Center for Epidemiologic and International Ophthalmology, University of British Columbia, Vancouver, Canada

**Keywords:** Blindness, diabetes mellitus, diabetic retinopathy, low vision, Yemen

## Abstract

**Background:**

We present a series of patients with diabetes mellitus (DM) who attended an eye hospital in Sana, Yemen during 2004.

**Aim:**

To determine the magnitude and risk factors of diabetic retinopathy (DR).

**Design:**

Cross-sectional study.

**Materials and Methods:**

Ophthalmologists assessed vision, ocular pressure, ocular media and posterior segment to note ocular manifestations among patients with DM. DR was graded by using bio-microscope and Volk lens. The prevalence and 95% confidence interval of ocular complications of DM were calculated. Risk factors of DR like age, sex, duration of diabetes and hypertension were evaluated.

**Statistical Analysis:**

Univariate and multivariate analysis.

**Results:**

Our series comprised 350 patients suffering from DM. The duration of diabetes was ≥15 years in 101 (29%) patients. Physician was treating 108 DM patients with insulin. The prevalence of DR was 55% (95% CI 49.6–60.1). The proportions of background diabetic retinopathy (BDR), preproliferative diabetic retinopathy (PPDR), proliferative diabetic retinopathy (PDR) and diabetic macular edema were 20%, 13%, 17% and 22% respectively. The prevalence of blindness among DM patients was 16%. The prevalence of cataract and glaucoma was 34.3% and 8.6%. Duration of DM was the predictor of DR. One-fifth of the patients had sight-threatening DR and needed laser treatment.

**Conclusions:**

DR was of public health magnitude among our patients. An organized approach is recommended to address DR in the study area.

Diabetic retinopathy (DR) is a priority disease in the ‘VISION 2020’ initiative for the global elimination of avoidable blindness. The World Health Organization (WHO) has recommended its member countries to integrate a program approach for DR within their prevention of blindness programs.[1] In industrialized countries, the magnitude of DR is high and it is the leading cause of blindness.[2] But countries with rapidly evolving economies and metros of developing countries also face the challenge of epidemic proportion of DR.[3,4] To our knowledge, no information on eye complications of diabetes in the Yemeni population has been reported in the past.

We treat patients of both the middle and lower middle-income groups in our institute. Eye examination with the help of modern instruments like indirect ophthalmoscope, bio-microscope, Volk lenses, applanation tonometer, autoperimeter, gonioscopes, etc is possible in our institute. Medical care services were provided at the diabetic center. This center has facilities to diagnose and manage Diabetes Mellitus (DM) and its systemic complications. The objective of our study was to estimate the magnitude and selected risk factors of eye complications of DM in our institution. On its basis, we also recommend a public health approach to address DR in the study area.

## Materials and Methods

The ethical and research committee of our university hospital approved this study. We obtained written consents from the patients to participate in this study. The procedures followed were in accordance with the ethical standards of the responsible committee on human experimentation (institutional or regional) and with the Helsinki Declaration of 1975, as revised in 2000.

This cross-sectional type of study was conducted from January 2004 to December 2004. The patients with DM attending ophthalmic clinic were our study population. These patients were referred by a physician for their eye examination. We included ‘1 to 6’ patients with diabetes on Saturdays, ‘7 to 12’ on Mondays, ‘13 to 20’ on Wednesdays. On an average the ophthalmologists used to see 20 DM patients in a day. The patients were sequentially enrolled and those giving consent to participate were examined as per protocol for this study.

One physician and two ophthalmologists who were experienced in treating DM and DR were our field investigators.

DM was defined as a person having fasting glucose level of ≥7 mmol/liter. If a patient was already taking medicine to control hyperglycemia, he/she was labeled as a case of ‘previously physician-diagnosed’ diabetes.[5] A medical history was obtained to determine the medical treatment being given and the duration of DM. The duration of DM was defined as the time interval in years between the date of first time diagnosis of DM and the date of present evaluation. A person was defined to suffer from hypertension if three repeated measurements at different instances in the day showed a reading of more than 140 mm Hg systolic blood pressure and/or >90 mm Hg diastolic blood pressure.[6] If patient was already taking medicine to control hypertension, he/she was labeled as hypertensive even though blood pressure measurements were within the normal range.

To assess the presence of systemic co-morbidities, the physician performed detailed clinical examination of cardio-vascular system, nervous system, renal system. Electro-cardiogram was obtained. Laboratory tests were performed for renal function tests and complete blood count and lipid profile. A person with diabetes who had clinically detectable albuminuria (≥300 mg/L) and did not have other renal disease explaining protein loss in urine was considered to suffer from diabetic nephropathy. If macrovascular late complications resulted in atherosclerosis in vessels of heart and patient had symptomatic coronary artery disease, the person was defined to have cardiac complication of diabetes. If a person suffered from symmetric distal and predominantly sensory polyneuropathy mainly resulting in stocking glove type of sensory loss, the person was considered to have complication of diabetic neuropathy.[7]

Ophthalmologists examined all these patients. Vision of each eye was noted with the best possible correction. Snellen's projection chart was used for this purpose. The WHO-recommended definitions of visual disabilities were adopted in our study. Blindness was defined as vision less than 10/200 in better eye after correction. Low vision was defined as vision less than 20/60 in better eye after correction. Anterior segment of each eye was examined using slit-lamp bio-microscope (Haag Streit). The ocular pressure was measured by an applanation tonometer (Zeiss). For patients suspected to have glaucoma, the field of vision was tested on Octopus automated perimeter. We asked patients to move their eyes in all eight directions to test the ocular mobility.

The pupils were dilated by instilling one drop of 1.0% tropicamide. If the pupil did not dilate after 30 min, we added one drop of 2.5% phenylephine to the previous one. The fundus was examined with +90 D Volk lens and bio-microscope. This enabled us to have a stereoscopic view of the retina and its vasculature. The presence and grading of DR was according to the International clinical DR and macular edema disease severity scale.[8] If exudates, microaneurysms and hemorrhages were present in retina but in an area other than the macula, we graded DR as background diabetic retinopathy (BDR). Presence of avascular zone and development of neovascular vessels was graded as preproliferative diabetic retinopathy (PPDR). If gliosis was present on the optic disc or along with blood vessels, the stage was graded as proliferative diabetic retinopathy (PDR). If the macula had edema or exudates with or without avascular zone, it was termed as diabetic maculopathy. If retinal detachment due to proliferation of glial tissue exerted traction, we graded the stage as tractional retinal detachment. If blood was present in vitreous and obscured details of the retina, we graded the stage as vitreous hemorrhage. We did not perform validity test and assumed that the diagnosis of two senior ophthalmologists adopting similar classification of DR would be similar.

The management of DR as recommended by the ophthalmologist was also evaluated. The WHO has given guidelines for laser treatment of cases with DR. We had used these guidelines for defining criteria for active intervention (laser treatment or surgery); in the rest of the cases primary prevention was recommended.[2]

A pre-tested form was used to collect the information for this study. The data was entered in an Excel Microsoft^®^ spreadsheet. It was checked for consistencies and duplications. For a univariate type of parametric analysis, we used Statistical Package for Social Studies (SPSS 11.5). Frequencies, percentage, their 95% confidence intervals were calculated. To compare the rates among variables, we used STATCALC of EPI6 info software to calculate Odd's ratio, its 95% confidence interval and Mantel-Henzal *P* value. To review the interaction of different risk factors, we carried out a binominal regression analysis. Presence or absence of DR was the dependent variable. Age, sex, duration of DM, hypertension and type of treatment for DM were the independent variables. A variable that did not have statistical significance was removed from the regression model.

All the cases with DR were explained about their ocular status and treatment was given at concession rates. DM patients with blindness or low vision disability were referred for rehabilitative services.

## Results

The physician and ophthalmologists examined 350 patients with DM in our study. The mean age when DM was first diagnosed in these patients was 44 years (Standard Deviation = 12.6 years). The mean age of patients with DR was 54.4 years (Standard Deviation = 11.8 years). One hundred and eighty-four (52%) patients had diabetes for more than 10 years. One hundred and eleven (31.7%) patients were taking injection insulin to treat their diabetes. Physicians noted that cardiac, cerebro-vascular, renal, peripheral vascular, neuropathy and other complications were present in 13, 8, 1, 2, 1 and 17 patients respectively. The mean duration of DM was 9.9 years (Standard Deviation = 7.77 years).

The prevalence of DR in our series was 55% (95% Confidence interval 49.6-60.1). The rate of DR by sex, duration, type of treatment and hypertension was calculated [Table 1]. The DR was significantly associated with the duration of DM (χ^2^ = 33.9, degree of freedom = 3, *P* < 0.001).

**Table 1:** Prevalence of diabetic retinopathy in Yemen

Variants	Examined	Diabetic retinopathy	Prevalence %	95% confidence interval	*P* value
Gender					0.31
Male	210	107	51.0	44.2 to 57.7	
Female	140	79	56.4	48.2 to 64.6	
Duration of DM					*P*<0.001
<5 years	113	21	18.6	11. 4 to 25.8	
5 to 9 years	53	26	49.1	35.6 to 62.5	
10 to 14 years	83	51	61.4	51.0 to 71.9	
1 5 and more	101	88	87.1	80.6 to 93.7	
Treatment					*P*<0.001
Diabetes managed without Insulin	239	104	43.5	37.2 to 49.8	
Diabetes managed with Insulin	111	82	73.9	65.7 to 82.0	
Hypertension*	81	50	61.7	51.1 to 72.3	
Total	350	192	54.9	49.6 to 60.1	

* Hypertension = (>140/90 mm Hg) without medication, dm = diabetes mellitus

We also compared the magnitude of DR in our patients with the rates reported in other studies in the Middle Eastern countries [Table 2].

**Table 2:** Diabetic retinopathy reported in Middle Eastern countries with Arab populations

Author	Year of publication	Sample size	Country	Rate %	Place of study
EI-Asrar AM, *et al.*[13]	1998	502	Saudi Arabia	31.3	Diabetes center
AI-Adsani AM[3]	2007	165	Kuwait	40	Diabetic clinic
AI-Till MI, *et al.*[18]	2005	986	Jordan	64.1	Hospital
AI-Maskari F, *et al.*[19]	2007	513	United Arab Emirates	19	Al Ain City
Khandekar R, *et al.*[20]	2003	2,249	Oman	14.5	Hospital
el Haddad OA, *et al.*[23]	1998	500	Oman	42.4	Hospital
Janghorbani M, *et al.*[21]	2003	549	Iran	8.9	Research center
Waked N, *et al.*[22]	2006	112	Lebanon	17	Research center
Herman WH, *et al.*[17]	1998	6052	Egypt	42.2	Hospital
Present study	2004	350	Yemen	54.9	Hospital

The patients with DM were categorized as no DR, BDR, PPDR and PDR. The rates of DR with macular edema and DR with rubeosis iridis were calculated separately. The number and percentage of DR cases and their 95% confidence intervals are given in [Table 3]. Bilateral serous macular edema was noted in 52 (14.9%) and unilateral serous macular edema was observed in 18 (5.1%) patients. Bilateral and unilateral ischemic macular edema were found in four (1.1%) and three (0.9%) patients respectively.

**Table 3:** Severity of diabetic retinopathy (DR) among patients with diabetes mellitus in DR study in Yemen

Diabetic retinopathy*	Number	%	95% confidence interval
Background diabetic retinopathy (BDR)	70	20	15.8 to 24.2
Preproliferative diabetic retinopathy (PPDR)	45	13	9.4 to 16.4
Proliferative diabetic retinopathy (PDR)	60	17	13.2 to 21.1
Diabetic macular edema	77	22	17.7 to 26.3
Rubeosis iridis	16	4.6	2.4 to 68
Not possible due to hazy media	5	1.4	0.2 to 2.7
No DR	147	42	49.6 to 0.1
**Total**	**350**		

* A person can have diabetic macular edema or rubeosis inais with or without having different stages of DR

The presence of co-morbidity in eye with less vision was taken into account to calculate magnitude of co-morbidity [Table 4]. In addition to DR, cataract and glaucoma were present in a significant number of cases with DM.

**Table 4:** Ocular morbidities in patients with diabetes mellitus in diabetic retinopathy study in Yemen

Morbidity	Number	%	95% confidence interval
Diabetic retinopathy	192	55	49.6 to 60.1
Cataract	120	34.3	29.3 to *39.3*
Glaucoma	30	8.6	5.6 to 11.5
Retinal detachment	16	4.6	2.4 to 6.8
Pseudo-exfoliation	12	3.4	1.5 to 5.3
Vitreous hemorrhage	15	4.3	2.2 to 6.4
Cranial nerve palsy	9	2.6	0.9 to 4.2
Central retinal vein occlusion	4	1.1	0.0 to 2.3
Uveitis	3	0.9	−0.1 to 1.8
Pterygium	4	1.1	0.0 to 2.3
Corneal pathologies	9	2.6	0.9 to 4.2
High myopia	8	2.3	0.7 to 3.9
Age-related macular degeneration	6	1.7	0.4 to 3.1
Disc Edema	3	0.9	−0.1 to 1.8

The rates of bilateral and unilateral blindness (VA < 10/200) were 56/350 (16%, 95% confidence interval 12.2–19.8) and 74/350 (21.1%, 95% confidence interval 16.9–25.5). The rate of Low Vision (VA < 20/60) was 194/350 (55.4%, 95% confidence interval 50.2–60.6).

The presence or absence of DR was the dependent variable. In the binominal regression model, we included age, sex, and duration of diabetes, and the mode of treating DM. The adjusted Odds ratio, 95% confidence intervals and *P* value are given in Table 5. Duration of DM was the predictor of DR in our study.

**Table 5:** Predictors of diabetic retinopathy among patients with diabetes mellitus in DR study in Sanna, Yemen

Risk factor	Adjusted odds ratio	95% confidence interval	*P* value
Age	0.98	0.96 to 1.00	0.11
Sex			
Male	1.28	0.46 to 1.30	0.33
Female	1.00		
Duration of diabetes mellitus			
<5 years	0.03	0.01 to 0.07	*P* < 0.001
5 to 10 years	0.13	0.06 to 0.30	*P* < 0.001
11 to 15 years	0.21	0.10 to 0.44	*P* < 0.001
15 years and more	1		
Hypertension			
Yes	0.74	0.40 to 1.38	0.34
No	1.00		

*The line of regression curve will cross ‘X’ axis at +16.64 with probability <0.001

In 74 (21.1%) patients, laser treatment was needed in one/both eyes, PRP in 13.4%, PRP with grid laser treatment in 15 (4.3%), treatment by laser with macular grid only in 12 (3.4%) patients.

## Discussion

The rate of DR among patients with DM in our study conducted in an eye unit of a hospital in the capital of Yemen was 54.9%. The rate of diabetic macular edema was 22%. The duration of diabetes was positively associated with the presence of DR.

The prevalence of DM was reported to be 4.6% and 9.75% in two different studies conducted in Yemen.[9,10] Risk factors such as obesity, impaired glucose tolerance, hypertension and hyperlipidemia exist in the Yemeni population.[9] Thus the burden of DM is likely to be significant and with improved socioeconomic conditions, especially in urban areas of Yemen, it is likely to further rise.[11] With improved health services, mortality due to renal and cardiac complications of DM will reduce and patients with DM would live longer.[12] DR that is associated with the duration of DM is therefore likely to increase.[13] Hence an organized approach to address DR within ‘VISION 2020’ initiative is now recommended.[14] For proper planning of a public health program, evidence-based information is crucial. Our study although hospital-based, provided information that would be useful for the Prevention of Blindness program of Yemen.

Our study had a few limitations. With a limited sample of patients visiting one institution, the results should be extrapolated with caution. The institution-based case selection could have introduced health-seeking bias. In addition, misclassification bias could have been introduced since the sample included a case mix of patients referred by physicians and patients presenting at the eye clinic directly.

The prevalence of DR (54.9%) among patients with DM in our study was high. Eye complications of DM in the Yemeni population are expected to be high because familial clustering and high rate of consanguinity are the genetic risk factors for DM which are reported to exist in the Yemeni population.[15,16] In addition, acquired factors like hypertension, obesity and hyperlipidemia are also reported to be common in this population.[9,10] A study in Yemen in 1997 had shown a rate of 45% micro-vascular complications in diabetic patients. [11] The patients in this study belonged to the manual labor class and this study did not represent the ‘well to do’ families of Sana city of Yemen. Risk of diabetes and eye complications of diabetes in this group are likely to be lower compared to the urban Yemeni population of our study. Hence comparison of the outcomes of this study with the results of the present study should be done with caution.

The rate of DR in our study was lower than that (64.1%) reported in Jordan.[12] This could be due to longer duration of DM and use of fundus fluorescein angiography (FFA) in the Jordan study. The mean duration of DM was 12 years in the Jordan study compared to 9.9 years in our study. In a study conducted in the United Arab Emirate (UAE), the prevalence of DR was 19%; much lower compared to our study.[13] Use of fundus photography to document the retinal changes of DR in UAE could be more precise. In Oman, the eye screening of patients with DM was introduced in 2000 while the diabetes control program through primary healthcare existed since 1990. The study showing 14.5% in Oman in 2003 could have been due to free and easy access to the health services to registered diabetics in Oman.[14] A study in Iran covered patients with non-insulin-dependent diabetes patients with a mean duration of diabetes of 6.9 years. This could explain the DR rate of 8.9% reported in the Ishfahan province of Iran.[15] A study in Lebanon that had 112 diabetic patients and retinopathy evaluation was conducted by using direct and indirect ophthalmoscopes reported 17% prevalence of DR.[16] In a study of diabetic patients in a hospital of Cairo, the prevalence of DR in diabetics was 42. 2%.[17] Thus it is evident that even in the Arab population of different Middle Eastern countries there was wide variation of DR. Different lifestyles, care for diabetes, time studies conducted and variation in tribes of Arab population could be the reasons for this variation.

The rates of DR were similar among males and females in our study. In contrast, Al Maskari *et al.,* and Khandekar *et al.,* reported higher rates of DR in males.[18,19] Less access of eye care to female patients in early stages of diabetes in Yemen could have resulted in this observation.

Duration of diabetes was positively associated with DR in our study. It was also noted in many other studies.[2,20,21] Early detection of diabetes through screening and regular follow-up and primary prevention is therefore recommended to reduce the risk of severe blinding complications of DR.

The prevalence of DR has been documented to be higher in Type 1 DM compared to Type 2 DM.[22] We had information on patients currently being treated with insulin and/or by other treatment modalities. We found that the rate of DR among patients using insulin was significantly higher compared to those using other medications. But long duration of DM and poor glycemic control could have prompted physicians to treat these cases with insulin. Thus association of type of treatment with DR could have been confounded by the duration and poor glycemic control in our study.

PDR was found in 17.1% of our patients. In Oman, El Hadad reported PDR in 12.8% patients.[22] High magnitude of PDR in our study could be due to lack of facilities for treating DR and poor compliance of patients for the laser treatment and for ignoring primary prevention of DM. It is interesting to note that the rate of maculopathy was 22% in our study. Al-Adsani reported 10.3% with maculopathy in Saudi Arabia and it was 5.1% in Oman. [3,14] In the absence of a large number of cases with nephropathy, we cannot explain the reason for a high rate of maculopathy in our study. The resources and skills required for treating maculopathy are different compared to the laser treatment for other stages of DR. The national programs aiming to offer care to cases of DR with maculopathy should plan accordingly.

The rate of un-operated cataract in patients with DM in our study was 34.3%. It was 38% in Jordan.[12] In the capital areas in both these studies, lack of eye care services could not be the reason for the backlog of cataract in patients with DM. Perhaps the criteria to operate cataract in DM patients in Jordan and Yemen might be different compared to those adopted for senile cataract cases.

Glaucoma was noted in 8.6% of patients with DM in our study. A study in Oman reported that 8.9% of patients with DM were suffering from glaucoma.[24]

Visual disabilities among diabetics are reported to be significantly higher compared to the general population.[25] The rates of bilateral blindness and unilateral blindness matched with a study in Jordan.[17] This information will be useful to the national planners of VISION 2020 in Yemen. Program approach adopted to address DR will reduce visual disabilities. The intervention strategies proposed for patients with DR in our study were primary prevention, prophylactic treatment by laser and management of macular complications. But awareness campaigns to improve regular follow-up and primary prevention have to be organized. Eye care services at an affordable cost should also be made available to all patients with DM.

## Conclusion

The prevalence of DR was high in our study. A public health approach is recommended to address eye complications of DR in the study area. Information on co-morbidities in the eye and visual disabilities among patients with DM should be further verified by studies with a larger sample and representing other parts of Yemen.
